# Novel Polymeric Nanocarriers Reduced Zinc and Doxycycline Toxicity in the Nematode *Caenorhabditis elegans*

**DOI:** 10.3390/antiox8110550

**Published:** 2019-11-14

**Authors:** Manuel Toledano, Manuel Toledano-Osorio, María D. Navarro-Hortal, Alfonso Varela-López, Raquel Osorio, José L. Quiles

**Affiliations:** 1 Faculty of Dentistry, Dental Materials Section, University of Granada, Colegio Máximo de Cartuja s/n, 18071 Granada, Spain; toledano@ugr.es (M.T.); mtoledano@correo.ugr.es (M.T.-O.); 2 Department of Physiology, Institute of Nutrition and Food Technology “José Mataix Verdú”, Biomedical Research Center, University of Granada, Avda del Conocimiento s/n., 18100 Granada, Spain; mdnavarro@ugr.es (M.D.N.-H.); alvarela@ugr.es (A.V.-L.); jlquiles@ugr.es (J.L.Q.)

**Keywords:** dentistry, doxycycline, fertility, growth, lethality, metabolism, nanoparticles, peroxide, toxicity, zinc

## Abstract

The objective was to evaluate the toxicity of zinc- and doxycycline-loaded polymeric nanoparticles (NPs) using *Caenorhabditis elegans* as a model organism. These NPs are composed of ethylene glycol dimethacrylate, 2-hydroxyethyl methacrylate and methacrylic acid. NPs were loaded with doxycycline (D-NPs) and zinc (Zn-NPs) by chemical adsorption, and loading efficacy was demonstrated. Worm death rate in a concentration-response curve basis was calculated for lethality. Metabolism was evaluated through pharyngeal pumping assay. Body length measurements, brood size and egg lays were used to gauge growth, reproduction and fertility respectively. Intracellular hydrogen peroxide levels were determined to assess the reactive oxygen species production. One-way ANOVA and Bonferroni were used for comparisons (*p* < 0.05). Tested NPs at the highest dosage did not affect lethality or worm metabolism, expressed in terms of death rate and pharyngeal pumping per minute, respectively. Zn-NPs slightly increased worm growth. The concentration of the intracellular hydrogen peroxide levels was the lowest in the D-NPs group. The distinct NPs and concentrations employed were shown to be non-toxic for in situ administration of zinc and doxycycline, reducing the harmful effects of these compounds.

## 1. Introduction

In organisms, the systemic distribution of conventional therapeutic agents diminishes the efficient dose of drugs reaching the target cells, and may cause not only toxicity but suboptimal results [1]. Nanotechnology is an actively developing discipline, and the growing use of nanoparticles (NPs) as drug carriers is producing rapid advances within this field [2]. The NPs’ large surface area per unit of mass and their reduced dimensions greatly facilitate their efficacy as drug carriers [2,3]. The short half-lives of many of the currently employed drugs have been a major motif for the use of drug delivery systems [4]. One of the main goals of drug delivery systems is to achieve the sustained effect of the drugs, reducing toxicity [5].

Drug delivery NPs may be categorized into two large groups: (i) degradable and (ii) non-degradable systems. When using degradable NPs, drugs are encapsulated into NPs and drug release depends upon NP degradation. All NPs will degrade at the same time. When water-soluble drugs are being transported, as its major release mechanism is diffusion control, a rapid drug release in a short period of time, known as ‘burst release’, is produced. Moreover, drug liberation is difficult to control, because NP degradation will be different with changes in temperature, pH, etc. [4]. Non-degradable NPs may be used instead. In this case, the drug is attached to the NP surfaces. However, NPs’ extensive usage in patients and consumers has increased the global concern of their potential adverse effects [6]. Thereby, some NPs may be toxic because of their size, their concentration, or their composition [3]. It has been determined that exposure to NPs leads to a significant reduction in lifespan [6]. 

Novel non-degradable (polymethylmethacrylate-based) polymeric spherical NPs have been synthetized. Polymethylmethacrylates are non-reabsorbable polymers, which have been used to clinically fix prostheses to bone due to their compatibility [7]. NPs are composed of 2-hydroxyethyl methacrylate as the backbone monomer, methacrylic acid as the functional monomer and ethylene glycol dimethacrylate as the cross-linker [8]. NPs are produced through polymerization precipitation. A Flory Hugings model-based thermodynamic approach using Hansen’s solubility parameters was used to control the process of precipitation. The basis of this model are the interactions of solvent molecules with growing polymeric chains by hydrogen-bonding, polar and dispersion forces [8]. 

Zinc and doxycycline are antibacterial [9,10] and potent matrix metalloproteinases (MMPs) inhibitors [11], they have also been shown to attenuate the oxidative stress [10,12,13,14]. However, even though zinc is essential for life, zinc may also cause toxicity in cells. This is generally exerted trough enzyme inhibition, cell membrane disruption, or by competing with other essential cations (i.e., Cu, Ca or Fe) [15,16]. Doxycycline is a potent broad-spectrum antibiotic that may also be toxic to healthy cells, through enzymatic inhibition or protein synthesis alterations [17]. Attempts to overcome doxycycline and zinc toxicity have led to the use of nanocarriers for delivery. Nanocarriers are able to localize the drug at the disease site, for a longer period of time, in a sustained manner, thus avoiding a rapid clearance through lymph drainage or even systemic toxic effects [17,18]. 

Therefore, zinc and doxycycline have been proposed for loading onto NPs, as they may decrease the toxicity of their free forms, and the NPs may facilitate a long-lasting drug release of up to 28 days [19,20]. The anionic carboxylate sequences (i.e., COO^−^) found along the polymeric NPs’ backbone may allow zinc quelation [19] and doxycycline binding through adsorption or by means of forming a peptide bond [20]. Therefore, Doxycyline-loaded NPs (D-NPs) and zinc-loaded NPs (Zn-NPs) have both been proposed as therapeutic agents in periodontal disease [20,21]. These NPs will facilitate the elimination of bacteria [20,21] and will avoid collagen hydrolysis by MMPs at the periodontal complex (bone, dental cement and periodontal ligament) [19,21]. Furthermore, mineral maturation and remineralization have been shown to occur after applying Zn-NPs and D-NPs on cervical dentin [22]. 

In periodontal defects, due to its anticollagenolytic effect, doxycycline potentiates the regeneration of hard tissues when it is administrated locally [23]. D-NPs reinforced the dentin microstructure, increasing cracking resistance [24,25], and promoting osteogenic factor release, including transforming growth factors, insulin-like growth factors, and bone morphogenetic proteins, which can promote the production of hard tissue [26]. However, doxycycline alters the conformation of MMP and renders them vulnerable to proteolytic digestion [27]. Then, it may not be suitable for all purposes, but there is a high chance that this wide action spectrum will alter other tissue repair processes profoundly, like the turnover of collagen [28]. In some cases, competitive inhibition of MMPs produced by zinc may be preferable [11]. Discrimination among clinical cases requiring D-NPs or Zn-NPs must be further elucidated trough clinical experiences. 

In ecotoxicological research, *C. elegans* is regularly employed as a study model for molecular to organismal ROS level responses, perceptible in cellular oxidative stress [2]. It has also been used to analyze other biological processes, such as metabolism, development, aging or retarded growth [29]. This animal has characteristics including transparency, small size (1 mm), short life (3–4 days at 20 °C), a more cost-effective experimental procedure, and high genetic and cellular similarity level with higher organisms [6,30]. *C. elegans* has the ability to provide nanomaterial information with respect to real-time toxicity at a subcellular, molecular and whole-organism level simultaneously, while subsequently aiding researchers in obtaining biosafety conclusions for more valuable nanomaterials [29]. *C. elegans* is composed of 959 somatic cells. Adult *C. elegans* possesses four organ systems, including the digestive, reproductive, immune and neuronal systems, the same as those in vertebrates. This allows *C. elegans* findings to be valuable and reliable [31]. *C. elegans* has been studied thoroughly with NPs. This model has an important advantage, as the nematode will ingest and incorporate the full NPs, giving an exact measurement of the NPs toxicity. In general, the majority of NPs display varying degrees of interference with *C. elegans* longevity, development, and reproduction [32]. Sensitive endpoints permit the detection of lower toxicant levels, may permit the employment of shorter testing intervals, and can offer understanding of the toxicants’ mechanisms of action [30,33]. An excessive amount of reactive oxygen species (ROS) production will produce early deterioration of reproductive organs in *Caenorhabditis elegans* [34]. This expression leads to cytotoxicity, inflammation, apoptosis and genotoxicity [6], or contributes to redox regulation and signaling. ROS is used as a generic collective term that indicates an active and reactive partially reduced number of O_2_ metabolites [35]. The body tolerates the ROS well only when generated in small amounts. Hence, exposure to any environmental stress will cause abnormally high ROS, which may lead to the appearancd of oxidative stress and some other various toxic reactions [36]. Oxidative stress happens when ROS level surpasses the endogenous antioxidant defense mechanisms capacity [13]. Even more, ROS may result in pathological periodontal tissue destruction [37], and may also hinder remineralization [38]. Zinc and doxycycline has been previously shown to be antioxidants [13,14], but it remains to be ascertained whether they may exert this action after being loaded onto NPs. 

The objective of this research was to assess the toxicity of zinc- and doxycycline-loaded NPs using *C. elegans* as a model organism. The null hypothesis tested is that lethality, metabolism, growth, reproduction, fertility and intracellular hydrogen peroxide levels of *C. elegans* will not be affected after exposure to not doped NPs, D-NPs and Zn-NPs.

## 2. Materials and Methods 

### 2.1. Nanoparticles Production

PolymP-*n* Active nanoparticles (NPs) were acquired (NanoMyP, Granada, Spain). NPs were synthetized through polymerization precipitation [8]. Hydrodynamic size distributions of NPs and polydispersity index were previously assessed by dynamic light scattering in deionized water. Mean sizes of NPs were as follows: undoped NPs (NPs) 250.1 ± 7.5 nm, Zn-doped NPs (Zn-NPs) 225.9 ± 8.9 nm and doxycycline-doped NPs (D-NPs) 244.4 ± 9.8 nm. NP sizes were not altered after loading, and there was no NP agglomeration. Polydispersity index of NPs was 0.05 ± 0.008 [39]. To proceed with the experiments, (1) undoped NPs (NPs), (2) (D-NPs), and (3) Zn-doped NPs (Zn-NPs) were suspended in phosphate buffered saline solution and tested at different concentrations.

### 2.2. Nanoparticle Characterization

5% *w/v* solutions of the polymeric spheres in distilled water were used. After drying in a vacuum heater during 24 h, polymeric spheres were analyzed by field emission scanning electron microscopy (FESEM) (GEMINI, Carl Zeiss SMT, Oberkochen, Germany) at 3 kV and 4.6 mm working distances.

Nanospheres were also examined by transmission electron microscopy (TEM) (LIBRA 120 PLUS, Carl Zeiss SMT). 

Additional characterization was performed by Atomic Forces Microscopy (AFM) (Nanoscope V, Digital Instruments, Veeco Metrology group, Santa Barbara, CA, USA). The images were undertaken in tapping mode, with a calibrated vertical-engaged piezo-scanner. A 10-nm-radius silicon nitride tip was attached to the end of the oscillating cantilever. This came into intermittent contact with the nanoparticle surfaces. Changes in the vertical position of the AFM tip at resonance frequencies near 330 kHz provided the height of the images registered as bright and dark regions. 

### 2.3. Loading Efficacy of Zinc and Doxycycline

For zinc-doping testing efficacy of NPs, 25 mg of NPs were submerged in 25 mL of eight different ZnCl_2_ aqueous solutions at concentrations ranging from 30 to 390 mg L^−1^. After 1, 2, 3, 4, 5, 6, 14 and 24 h, the suspensions were submitted to centrifugation (7800 rpm/G-force = 6461) for 20 min and the particles were separated from the supernatant. Supernatants were stored until testing (−20 °C). Zinc concentrations were assessed through an inductively coupled plasma optical emission spectrometer (ICP-OES Optima 8300, Perkin-Elmer, Waltham MA, USA). The ion complexation values for each ZnCl_2_ solution and time point were obtained by subtraction of the final zinc content in supernatants from the initial zinc content in ZnCl_2_ solutions, before NPs incubation. Experiments were performed in triplicate [20]. 

For doxycycline-doping of NPs, 25 mg of NPs were immersed in 25 mL of doxycycline hyclate aqueous solution (Sigma Aldrich, Chemie Gmbh, Schnelldorf, Germany) at eight different concentrations ranging from 70 to 870 mgL^−1^, during 10, 20, 30 and 90 min under constant shaking. Subsequently, suspensions were submitted to a centrifugation process (7800 rpm/G-force = 6461) for 20 min. NPs were separated from the supernatant. Until testing doxycycline concentration, supernatants were stored (−20 °C). The amount of doxycycline was assayed using high-performance liquid chromatography (HPLC) (Waters Alliance 2690, Waters Corporation, Milford, MA, USA) equipped with a UV-Vis detector. A binary mobile phase consisting of solvent systems A and B was used in an isocratic elution with 80:20 A:B. Mobile phase A was 50 mM KHPO_4_ in distilled H_2_0 and mobile phase B was 100% acetonitrile. The HPLC flow rate was 1.0 mL/min and the total run time was 10 min. The retention time was 4.85 min. The concentration of doxycycline was calculated based on a standard curve of known levels of doxycycline at 273 nm. Doxycycline loading values on NPs were calculated by subtraction of the final doxycycline content in supernatants from the initial doxycycline content in solutions, before NP incubation. Experiments were performed in triplicate [20].

### 2.4. Test Organism

Strains of *Caenorhabditis elegans* wild type N2 and transgenic JV1 (jrIs1 [rpl-17p: HyPer + unc-119(+)]) were supplied by the *Caenorhabditis Genetics Center* (CGC) (Minneapolis, MI, USA) for this study. N2 Bristol and JV1 were routinely maintained in an incubator (VELP Scientifica FOC 120 E, Usmate, Italy) at 20 °C on plates with nematode growth media (NGM) containing an *E. coli* OP50 lawn, as previously described [40]. All tests were conducted starting with egg-synchronized worms with bleaching solution (0.5 N NaOH in 20% bleach).

### 2.5. Lethal Toxicity Test

A lethality assay was carried out in N2 strain to determine the acute toxicity of the studied NPs by evaluating the death rate in a concentration-response curve basis. N2 Bristol age synchronized larvae L4 were transferred to NGM plates without food source. A concentration-response curve was prepared for each type of NP. The animals were exposed to increasing concentrations of the NP dissolved in NGM (0, 1, 5, 10 mg/mL) for 24 h at 20 ± 1 °C. The animals were scored as live or death using a Motic dissecting microscope (Motic Inc., LTD., Hong Kong, China). Death was assumed when there was no response to mechanical stimulus generated with a platinum wire [41]. Three independent assays for each NP type and concentration value were made. Each independent assay included three NGM plates with ten worms in each one. A total of 90 worms were then tested for each experimental group. Lethality was expressed as a total percentage of worm survival. Lethality test results were used to choose the concentration for each treatment (i.e., NPs, Zn-NPs or D-NPs) in subsequent assays.

### 2.6. Metabolism: Pharyngeal Pumping Assay

Worm metabolism was assessed through pharyngeal pumping assay by measuring grinding movement of the pharynx in N2 strain [42]. For this purpose, worms in the larval stage 4 (L4) were incubated at 20 °C in NGM plates with 10 mg/mL of the different NPs prepared as in the previous assay. After 24 h of exposure, the plates were rinsed with phosphate buffered solution to collect the nematodes. On different plates with NGM, nematodes were placed with *E. coli* to count the terminal pharynx bulb contractions number per minute with a Motic microscope (Motic Inc., LTD. Hong Kong, China). A minimum of ten worms were studied per experiment (*n* = 10) and the experiment was performed in triplicate.

### 2.7. Body Length: Growth Test

The body length measurement was employed to assess the treatments effect on the worm development. N2 eggs, acquired from gravid adults by bleaching, were placed on three NGM plates, each containing a different type of NPs (NPs, Zn-NPs or D-NPs) at 10 mg/mL and one plate without NPs was also tested, all planted with *Escherichia coli* OP50 as a source of food. The plates were incubated at 20 °C over five days. After that time, five-day-old adults were photographed on a Motic dissection microscope (Motic Inc., LTD. Hong Kong, China), and their body lengths were measured [43] using Motic Images Plus 3.0 (Motic Inc., LTD. Hong Kong, China) from pictures. A minimum of one hundred worms were measured per treatment. Results were showed as percentage of body length variation with respect to the control group.

### 2.8. Reproduction and Fertility Asays

NPs’ effects on reproduction and fertility of nematodes were assessed using brood size and egg-laying capability, respectively. Synchronized L4 stage N2 worms were placed on NGM plates prepared as in the previous test. Animals were submitted to experimental solutions for 24 h incubated at 20 °C. Each worm was moved to an untreated NGM well with an *E. coli* OP50 lawn inside a 24-well plate. This step was repeated daily until the hermaphrodite stopped of laying eggs. Egg and hatched larvae numbers per well were counted using a Motic microscope dissection (Motic Inc., LTD. Hong Kong, China) the same day of adult removal and 24 h after, respectively [44]. A minimum of twenty-four worms were studied per treatment. Additionally, fertility (egg number) and reproduction (larvae number) were assessed at intervals between 24 and 120 h, and 48 and 144 h, respectively.

### 2.9. ROS Production

Real-time H_2_O_2_ production in the JV1 strain was explored following the methodology of Back et al. [45] with variations. The JV1 strain genetically expresses Hyper, a hydrogen peroxide-specific sensor. This sensor consists of a yellow fluorescent protein introduced into the H_2_O_2_-sensitive regulatory domain of the bacterial transcriptor factor OxyR (OxyR-RD). HyPer selective and sensitive oxidation by H_2_O_2_ generates a disulfide bridge between the OxyR-RD separated parts, subsequently altering the protein’s fluorescent properties [46]. Briefly, for the micro plate reader quantification of H_2_O_2_ production, synchronized eggs were exposed to experimental treatment for 48 h, then 250 pelleted L4 worms were located in a black, flat-bottom 96-well microliter plate (Greiner, Madrid, Spain). Fluorescence was assessed at 25 °C for 15 min with a Synergy Neo2 micro plate reader (Biotek, Winooski, VM, USA.) with excitation filters of 490 nm (oxidized) and 405 nm (reduced), and an emission filter of 535 nm. Baseline and H_2_O_2_ (20 mM H_2_O_2_)-induced levels were calculated. All data are the average of three different experiments. All measurements were performed in duplicate. Each technical fluorescence repetition was normalized to the real number of tested worms after counting under a Motic SMZ-143 stereomicroscope (Motic, Hong-Kong, China). ROS production was obtained by calculating the ratio of the 15 min average intensity of fluorescence at 490 nm excitation and 535 nm emissions divided by the 15 min average intensity of fluorescence at 405 nm excitation and 535 nm emissions. For the anatomical localization of H_2_O_2_ production, confocal microscopy was used. We used a Nikon A1r confocal laser scanning microscopy system mounted on a Nikon Ti-E inverted epifluorescence microscope (Nikon Instruments Inc., Melville, NY, USA.), equipped with a 405 nm diode laser and a 488 nm multiline Ar-laser. After treatment with 20 mM H_2_O_2_ or without any treatment (to test baseline internal H_2_O_2_ production), worms were immobilized in an appropriate coverslip by adding 10 µL of sodium azide and scanned with a Plan Fluor 10×/0.5 objective. Worms were sequentially excited with both laser lines, set a minimal intensity to avoid ROS induction and bleaching, and detected through a 525/50 nm band-pass filter. Images were acquired and handled with the NIS-Elements 3.2 software (Nikon Instruments Inc., Melville, NY, USA.).

### 2.10. Statistical Analysis

Variables were studied for normality (Kolmogorov-Smirnov) and homogeneity of variance (Levene). Prior to analysis, non-normally distributed variables were transformed. One-way ANOVA and Bonferroni were employed as post hoc tests. Significance was considered for *p* < 0.05. Statistical analysis was performed by IBM SPSS 24.0 (IBM Corporation, Armonk, NY, USA).

## 3. Results

### 3.1. Nanoparticle Characterization 

**Figure 1:**
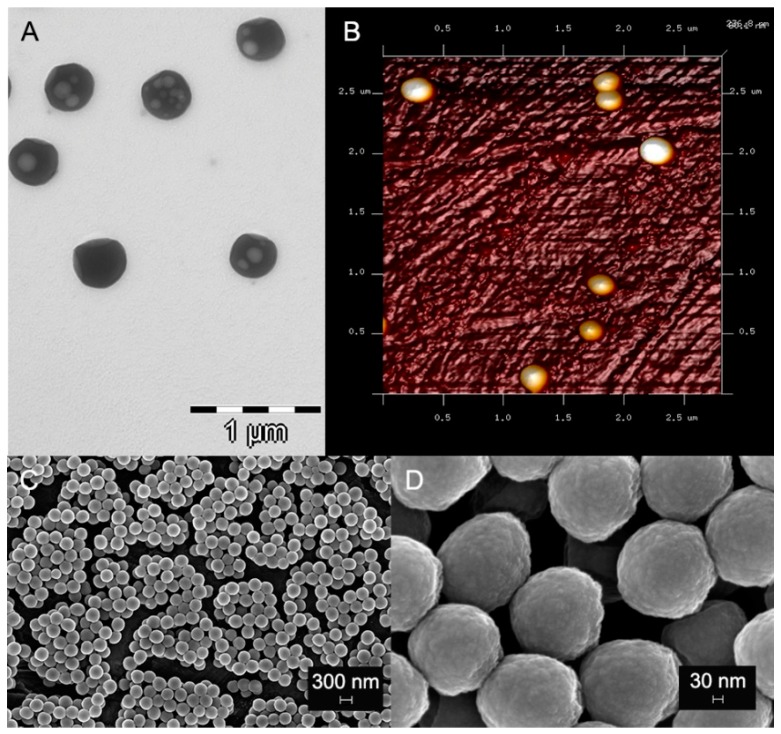
Images of nanoparticles observed under TEM (**A**), AFM (**B**) and FESEM (**C** and **D**). NPs are spherical and homogeneous in shape. NPs do not agglomerate, and they are approximately 250 nm in diameter.

The obtained images are displayed in Figure 1. Figure 1A presents TEM images, Figure 1B displayed AFM pictures and Figure 1C,D shows NPs observed under the FESEM. NPs did not agglomerate, and presented a spherical and homogeneous shape. They are non-porous NPs. Light circular objects inside of the NPs in TEM images (Figure 1A) were artifacts developed during electron beam transmission. 

### 3.2. Loading Efficacy of Zinc and Doxycycline

**Figure 2:**
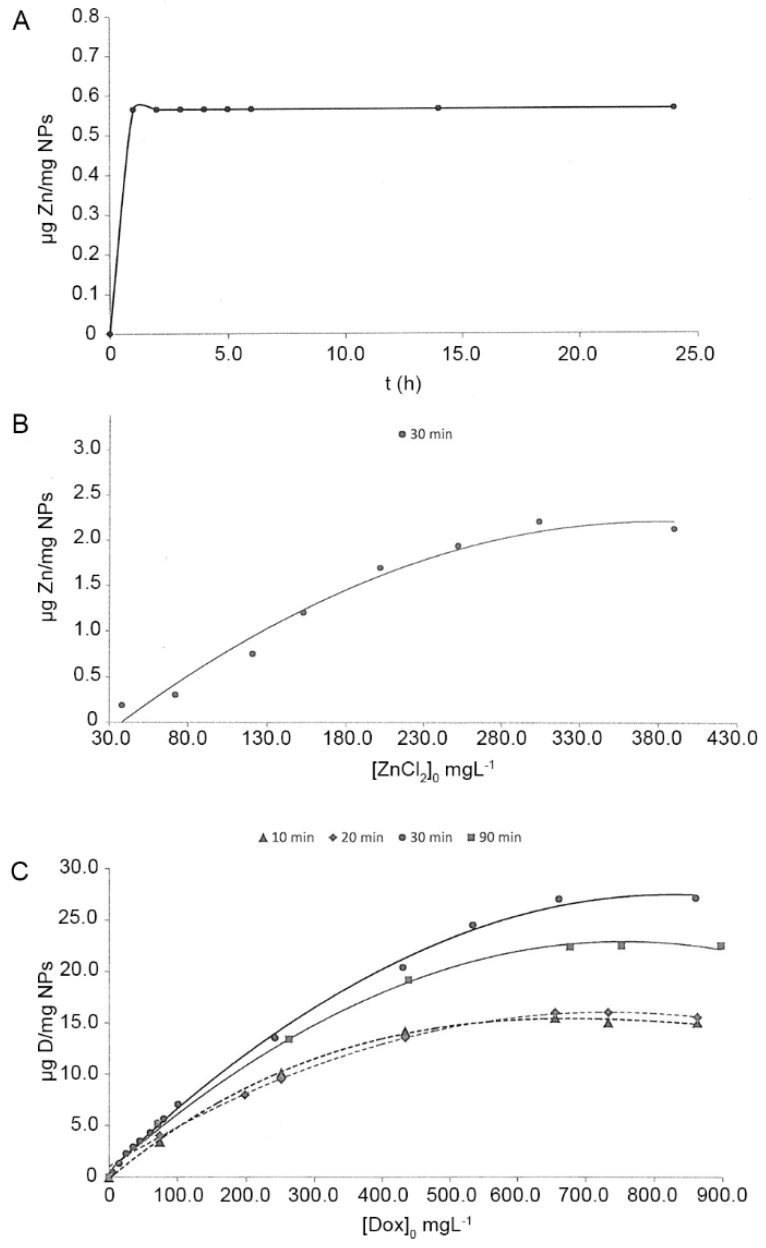
Zinc adsorption kinetics on nanoparticles as a function of time (**A**), Zinc adsorption kinetics on nanoparticles as a function of zinc concentration in initial solutions (**B**) and doxycycline adsorption kinetics on nanoparticles as a function of time and doxycycline concentration in initial solutions (**C**).

The attained absorption equilibrium of zinc as a time function and ZnCl_2_ solution concentrations is shown in Figure 2A,B. The maximum zinc complexation value (2 µg Zn/mg NPs) was attained after the NPs’ immersion in ZnCl_2_ solution, at a concentration of 300 mg L^−1^, for 90 min. Doxycycline doping values for NPs are displayed in Figure 2C. The maximum doxycycline adsorption value was achieved after 30 min of NP incubation, which corresponded to 27.1 µg Dox/mg NPs using a solution of doxycycline hyclate at a concentration of 750 mg L^−1^. 

### 3.3. Lethal Toxicity Assay

**Figure 3:**
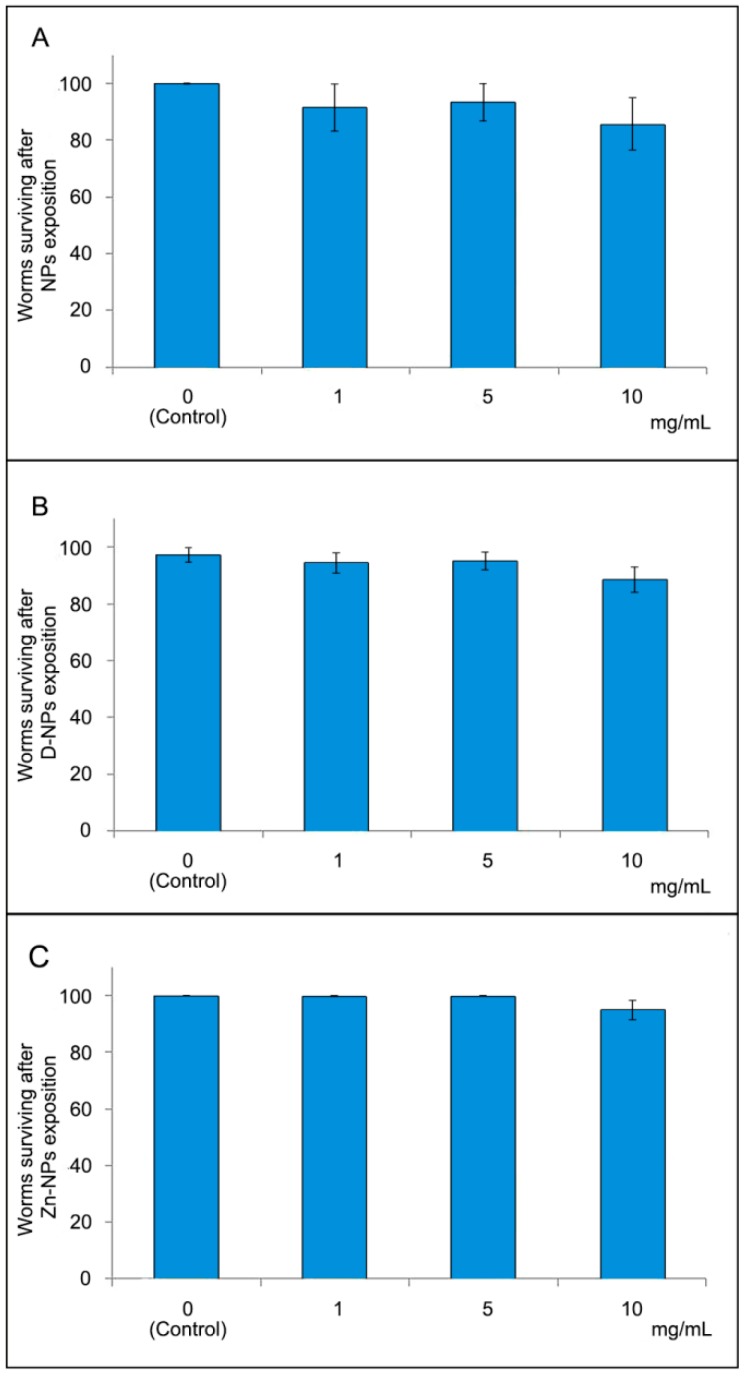
Lethal toxicity expressed as a percentage of surviving worms, after the nematodes were exposed to different nanoparticles and concentrations. Results are presented as mean and standard error of three different assays (**A–C**). Each independent assay included three NGM plates with ten worms in each one. A total of 90 worms were then tested for each experimental group. No differences in worm surviving percentages were found after ANOVA analysis (*p >* 0.05). D-NPs: doxycycline-doped nanoparticles. NPs: undoped nanoparticles. Zn-NPs: zinc-doped nanoparticles.

The results of the lethality assay can be observed in Figure 3. Between groups, no differences in survivorship were observed, irrespective of NPs type or dosage (*p* > 0.05).

### 3.4. Pharyngeal Pumping Assay

**Figure 4:**
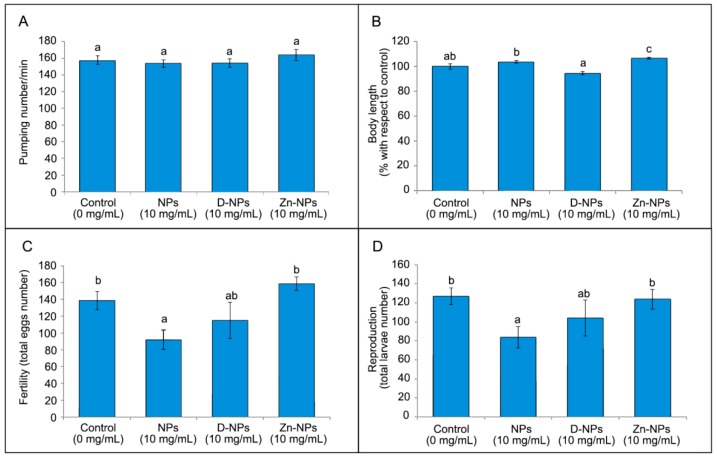
Mean and standard error of pharyngeal pumping (**A**), body length (**B**), fertility (**C**) and reproduction (**D**) of *Caenorhabditis elegans* exposed to different NPs, at 10 mg/mL concentration. One-way ANOVA was not significant for pharyngeal pumping (*p* > 0.05). ANOVA tests were significant for body length, fertility and reproduction (*p* < 0.05). For each graph, similar letters (a,b) indicate no significant differences between the NPs experimental groups, after Bonferroni post hoc testing at *p* < 0.05. D-NPs: doxycycline-doped nanoparticles. NPs: undoped nanoparticles. Zn-NPs: zinc-doped nanoparticles.

The metabolism assay expressed in terms of pharyngeal pumping per minute is presented in Figure 4A for the three treatments assayed. No differences were found in metabolism between control group and any of the three assayed NPs (*p* > 0.05).

### 3.5. Growth Assay

Concerning growth testing by body length measurement. Zn-NPs (10 mg/mL) produced significantly higher growth (*p* < 0.05) than the control group (Figure 4B). 

### 3.6. Reproduction and Fertility Assays

Reproduction and fertility assays were realized to evaluate the progeny production of treatment and control groups, counting total larvae and total eggs, respectively. *C. elegans* treated with unloaded NPs reduced both total egg number (fertility assay) and total larvae number (reproduction assay) if compared to the control group (Figure 4C,D).

### 3.7. ROS Production

**Figure 5:**
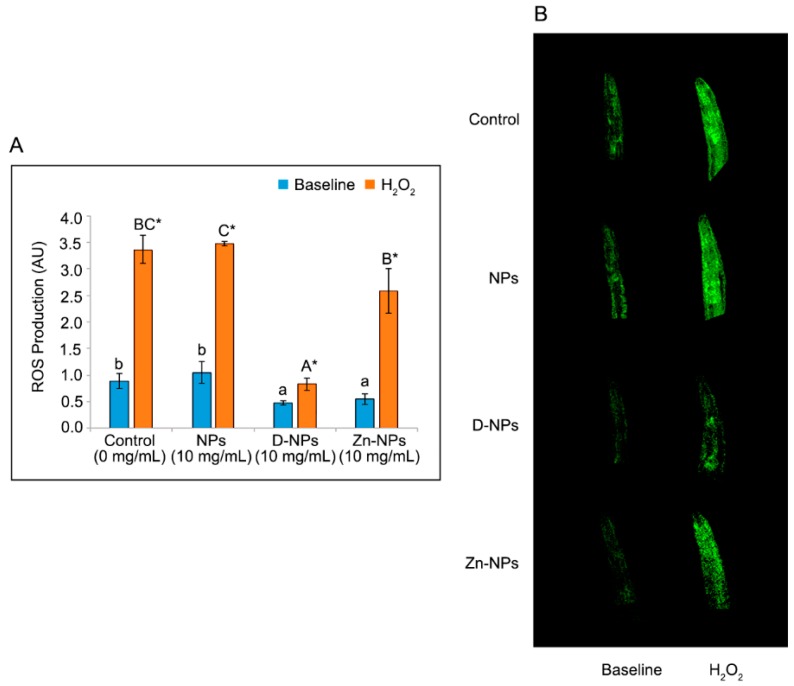
Intracellular hydrogen peroxide production, expressed as the ratio of in oxidized/reduced Hyper, when *Caenorhabditis elegans* is exposed to the different nanoparticles at 10 mg/mL concentration. Results are presented as mean and standard error. Different lowercase letters (a,b) indicate significant differences (*p* < 0.05) between the distinct experimental groups at base line. Different capital letters (A–C) represent the existence of significant difference (*p* < 0.05) between the experimental groups induced with 20 mM of hydrogen peroxide. The asterisk (*) represents the existence of statistically significant differences (*p* < 0.05) between the baseline and the induced with hydrogen peroxide levels within the same experimental group (**A**). Confocal microscopy images (5×) representing worm’s fluorescence at same dosages of NPs and H_2_O_2_ as for Figure 5A D-NPs: doxycycline-doped nanoparticles. NPs: undoped nanoparticles. Zn-NPs: zinc-doped nanoparticles (**B**).

ROS production by worms is presented in Figure 5. Concerning baseline levels, the highest values were found for control and NPs groups, with no differences between them but with higher values than those found for D-NPs and Zn-MPs treated worms. To test maximal susceptibility to oxidation, the experimental groups were subjected to an external induction with H_2_O_2_. The results of inducted ROS showed that, for all groups, higher ROS data were found with respect to their baseline counterparts. Concerning comparison between groups for ROS induced levels, the lowest values were found for worms treated with both D-NP, followed by those treated on Zn-NPs.

## 4. Discussion

The main outcome of this research was that none of the tested NPs affected either lethality or worm metabolism, expressed in terms of death rate and pharyngeal pumping per minute, respectively. Un-doped NPs slightly reduced fertility and reproduction. However, Zn-NPs increased worm growth. Zn-NPs and D-NPs reduced the concentration of the intracellular hydrogen peroxide levels if compared to the control group.

The *C. elegans* main strength is their short lifespan, small size, and low cost, which define this nematode’s adaptability to laboratory and chronic exposure studies. *C. elegans* could be a useful assay model to evaluate NPs toxicity or NPs beneficial functions in living bodies, due to the high sensitivity of nematodes to any environmental toxicant [31].

The digestive tract is the way of entry of NPs into the body of *C. elegans* [31]. The size of NPs should be considered when testing toxicity mechanisms using the present model. It has been previously described that the pharynx and the buccal cavity of *C. elegans* exclude large particles. Particles of a size range between 0.5 and 3 μm are ingested by *C. elegans* [47]. This range includes the size range of bacteria consumed by *C. elegans* in its natural habitats, as it does *E. coli*, a rod-shaped bacterium, measuring approximately 0.5 μm in width by 2 μm in length (2000 nm). Then, present NPs (250 nm) will be ingested by nematodes without problems of size exclusion. 2 µg Zn/mg NPs and 27.1 µg Dox/mg NPs were loaded onto NPs after immersion in ZnCl_2_ and doxycycline hyclate solutions (Figure 2C). This model has an important advantage for toxicity testing as the nematode will ingest the full NPs, giving an exact measurement of the loaded NPs toxicity. Then, the liberation kinetics will not affect the attained results.

Mortality test assessment is most frequently performed to determine the acute toxicity of the NPs by generating a concentration-response curve for *C. elegans* [31]. *C. elegans* lethality was not influenced by the different NPs at any of the tested concentrations (Figure 3). Although lethality in toxicology is regarded as the most common and basic endpoint, its inadequate sensitivity negatively influences the accuracy and increases the errors in experimental determination [30]. Pharyngeal pumping speed, related with defecation cycle length, is an indicator commonly employed to study NPs’ effect on the alimentary canal. Hence, the metabolism assessed showed that no differences among groups appeared in the pharyngeal movement (Figure 4A), which is metal-specific in *C. elegans* [33]. It was observed that particles’ toxicity is not just linked to composition, but also to their sizes. Generally, smaller sized particles are supposed to develop greater toxicity [48,49].

Zinc, as a therapeutic agent, can be used in different presentations, e.g., as free ionic forms (Zn^2+^), in an oxidized form (ZnO), or quelated to polymeric NPs (Zn-NPs), as in the present study. Metals free ionic forms are generally considered to be more toxic to biota than precipitated and complexated forms [49]. ZnO-NPs exposure has been well documented as a serious human health and environment threat. ZnO-NPs have been shown to induce damage to mitochondria and chromosomes [2,50], they may also inhibit important enzymes activity and may disrupt the cell membrane integrity [49]. The present Zn-NPs contain about 2 µg of Zn per mg of NPs (Figure 2B) and were used at a concentration of 10 mg per mL. Taking this into account, we know that 0.02 mg/mL of Zn is being applied to *C. elegans*, which is a much higher concentration than that used in the study of Sonane et al. [6]. Moyson et al. also obtained a mortality rate increase in Zn-exposed nematodes when using 0.005 mg/mL of Zn, diluted at 200 and 400 mg/L after 2 h [51]. It is, then, important to highlight that when zinc is administered to nematodes doped on the present NPs carriers (even at higher doses than those of previous studies -up to 0.02 mg/mL), it is non-toxic, as no differences were found between the Zn-NPs exposed nematodes and the non-Zn-exposed nematodes survival from the control group (Figure 3).

A very sensitive way to test treatment toxicity in *C. elegans* is the analysis of fertility and reproduction. Lower concentrations of tested agents than the concentrations that may affect *C. elegans* behavior and viability, usually alter reproduction and fertility [31]. In the present study, *C. elegans* treated with unloaded NPs slightly reduced (by about 17%) both fertility and reproduction when they were compared with the control group (Figure 4C,D). The null effect of NPs on the *C. elegans* lifespan implies that it is the NPs’ short-term influence and will not cause permanent damage to the organisms. This suggests that specific subcellular process disturbances were not found to be linked to inhibition and lifespan reduction development [32]. Therefore, none of the NPs suppressed the reproductive process in *C. elegans*, implying no toxicity or different effects on worms’ reproduction. Nevertheless, the worms may have been affected by the undoped NPs through the sequential development period, including spermatogenesis, gonadogenesis and oogenesis by inducing aging of the reproductive system [52]. It is also important to consider that *C. elegans* has the capacity to adapt or acclimate to the presence of NPs across multiple generations [31].

The nematode’s body length (i.e., growth) can be considered a development measurement. It is usually employed as a marker of toxicity because it has been previously stated that exposure to NPs can postpone nematode development at the first or second larval stages [41]. Then, the animals’ body length measurements are employed as an indicator of degree of growth inhibition [32]. Body length growth inhibition was not encountered in the present research; moreover, Zn-NPs promoted a significant increase in growth when compared to the control group (Figure 4B). This might be a consequence of the MMPs inhibitor characteristic that Zn-NPs may develop [19]. Zn-NPs also affect molecular signaling pathways and stimulate both different metabolic effects in distinct organisms [53] and innate immune systems [54]. Zinc has also been shown to improve both weight and height development in humans [55]. Therefore, Zn-NPs favored a growth increase. It is remarkable that in a different study, in which 0.02 mg/mL of Zn in a free form were applied to nematodes, a 55% inhibition in both body length growth and worm reproduction were encountered [30].

With respect to the effects of doxycycline on the growth of worms, it is known that this molecule induces a significant delay of growth, another well-known physiological effect caused by the antibiotic [56]. In our study, D-NPs attained similar values of body length to the control group. Doxycycline may act as an inhibitor of mitochondrial translation, reducing oxygen consumption and metabolic rates [57,58]. In this respect, a little number of mitochondrial genomes are enough to support life, but more genomes are required for energy intensive processes such as development and reproduction [59]. Our drug delivery systems’ efficacy can be related to their size and chemical composition, causing the reduction of the drug toxicity [60]. In the present research, the D-NPs contained 271µg of doxycycline per mg of NP, and were used at a concentration of 10 mg per mL (Figure 2C). Therefore, 2.710 mg/mL of doxycycline were really being applied to *C. elegans*, which is a higher concentration than that showing toxicity in the study of Tsang and Lemire [59]. These authors reported a dose-dependent delay of larval growth with doxycycline at concentrations ranging from 60 to 120 µg/mL (0.06 and 0.12 mg/mL respectively). Therefore, considering the low toxicity attained and the high doxycycline concentration that was administered to the worms, the use of the present D-NPs is encouraged.

Oxidative stress is considered to be the main mechanism by which NPs cause toxicity. In other studies, accumulation of reactive oxygen species (ROS) is usually observed in *C. elegans* treated with NPs [31,52]. This may result in increased lethality, decreased body growth, altered development, intestinal impairment, and reproductive affectation in a concentrated manner. Therefore, ROS production is studied, as it can be a highly sensitive endpoint used for bio-safety assessment of specific NPs in *C. elegans* [31]. In the present study, any of the tested NPs produced ROS accumulation, even when oxidative stress damage presents the prime mechanism causing toxic effects after testing other different nanoparticles [31,52]. This clearly indicates that ROS production may be specific and will not only depend on NP size, but also on NP chemical composition.

A ROS reduction, different from the non-exposed control group, was found in nematodes exposed to D-NPs and Zn-NPs (Figure 5). Results concerning the ROS reduction attained by Zn-NPs are in agreement with those results from Marreiro et al. [14], who stated that zinc reduced hydroxyl radicals and sequestered ROS produced in stressful situations. This would cause a reduction of the oxidative damage to the worms, hence delaying aging and improving the nematodes’ lifespan [30]. The antioxidant activity shown by D-NPs (Figure 5A,B) may be explained by the scavenging capabilities of doxycycline’s phenol ring. It is presumed that the reaction of phenol ring and ROS produces an unreactive and relatively stable phenol radical, due to the resonance stabilization and steric hindrance caused by the phenol ring side groups ring [13]. Furthermore, mitochondria are protected by doxycycline against fragmentation induced by ROS and prevented the collapse of the mitochondria membrane potential processes caused by ROS. That would produce rapid cellular and mitochondrial function impairment causing necrotic and apoptosis cell death [10]. Attained ROS reduction by D-NPs and Zn-NPs may be particularly useful when treating periodontal disease [61].

Present NPs may not only be useful for carrying zinc and doxycycline, but also any other metal ions, such as magnesium, calcium, strontium or even any protein that will be easily conjugated to the carboxyl groups located at the NPs surfaces. Future studies will need to evaluate any long-term effects of the tested NP applications. The next steps in the present research include the application of noninvasive X-ray absorption near edge structure spectrometry (XANES), electron probe microanalysis (EPMA), and nuclear microprobe (NMP), as new platforms for studying interactions between living *C. elegans* and NPs. Research on Endoplasmic Reticulum Stress of present NPs using *C. elegans* model is also encouraged for the future [31,62].

## 5. Conclusions

The distinct NPs at the concentrations used in the present study did not affect lethality neither pharyngeal movement of the worms. Zn-NPs favored a growth increase. D-NPs and Zn-NPs produced lower reactive oxygen species (ROS) activity and thereby minor stress reaction and oxidative damage to the *Caenorhabditis elegans*. The novel NPs presented may be proposed for in situ administration of zinc and doxycycline, due to the null harmful effects and the antioxidant ability of these compounds.
